# Development of quality indicators for hand osteoarthritis care – Results from an European consensus study

**DOI:** 10.1016/j.ocarto.2025.100578

**Published:** 2025-02-05

**Authors:** Daniel H. Bordvik, Yeliz Prior, Rachael Bamford, Francis Berenbaum, Mathilda Björk, Thalita Blanck, Barbara Slatkowsky Christensen, Krysia Dziedzic, John Edwards, Nazemin Gilanliogullari, Carol Graham, Ida K. Haugen, Margreet Kloppenburg, Hellen Laheij, Marco J.P.F. Ritt, Tanja Stamm, Anne Therese Tveter, Nina Østerås, Ingvild Kjeken

**Affiliations:** a Center for Treatment of Rheumatic and Musculoskeletal Diseases (REMEDY), Diakonhjemmet Hospital, Oslo, Norway; b Rehabilitation West A/S, Haugesund, Norway; c Centre for Human Movement and Rehabilitation, School of Health and Society, University of Salford, Salford, UK; d Nottingham CityCare Partnership CIC, Nottingham, UK; e Department of Rheumatology, Sorbonne University, INSERM, APHP Saint-Antoine Hospitalier, France; f Pain and Rehabilitation Centre, Department of Health, Medicine and Caring Sciences, Unit of Occupational Therapy, Linköping University, Linköping, Sweden; g Impact Accelerator Unit, School of Medicine, Keele University, UK; h European University of Lefke, Faculty of Health Sciences, Department of Physiotherapy and Rehabilitation, Lefke, Northern Cyprus, TR-10 Mersin, Turkey; i Rheumatology Occupational Therapy Service, Haywood Hospital, Midlands Partnership University NHS Foundation Trust, Stoke-on-Trent, Staffordshire, UK; j Department of Rheumatology and Clinical Epidemiology, Leiden University Medical Center, Leiden, the Netherlands; k Leiden University Medical Center, Leiden, the Netherlands; l Department of Plastic, Reconstructive and Hand Surgery, Amsterdam UMC, the Netherlands; m Institute for Outcomes Research, Center for Medical Data Science, Medical University of Vienna, Ludwig Boltzmann Institute for Arthritis and Rehabilitation, Vienna, Austria; n Institute of Rehabilitation Science and Health Technology, Oslo Metropolitan University, Oslo, Norway

**Keywords:** Hand osteoarthritis, Quality indicators, Patient-reported, Care, Health services

## Abstract

**Background:**

People with hand osteoarthritis (OA) often have poor access to recommended treatments. To enhance care quality, quality indicators (QIs) based on clinical recommendations are essential. Current QI sets, like the Osteoarthritis Quality Indicator Questionnaire (OA-QI v.2), primarily address hip- and knee OA, and not hand OA.

**Objectives:**

To adapt the OA-QI v.2 for assessing patient-reported quality of hand OA care.

**Design:**

We used the OA-QI v.2. set as a starting point and adapted it to reflect hand OA care. A literature search was performed to identify potential QIs for hand OA following the Rand/UCLA Appropriateness method. A European expert panel, comprising researchers, clinicians, and patient research partners, participated in online meetings to discuss adaptation and suggest new QIs based on treatment recommendations for hand OA, and anonymously rated each suggested QI regarding its importance, validity, usefulness, and feasibility. Consensus was defined by predefined rating cut-off scores. The adapted questionnaire was translated from English into Norwegian. Cognitive debriefing interviews with Norwegian and UK hand OA patients were conducted to ensure clarity.

**Results:**

Our initial literature search provided 1670 articles, with none describing relevant QIs. After three voting rounds, sixteen QI items reached consensus, reflecting current hand OA care standards. Items were generally well understood, requiring only minor clarity amendments after patient interviews (N ​= ​28).

**Conclusion:**

The OA-QI v.2 was successfully adapted into a 16-item Hand OA-QI set ensuring alignment with international care standards for hand OA through literature review, international expert panels and patient feedback on language and layout.

## 1 Introduction

Hand osteoarthritis (OA) is a prevalent joint disease that contributes significantly to the global burden of pain, disability, and reduced health-related quality of life [1]. The lifetime risk of developing hand OA approximates 40 ​% [2]. There is yet no cure for hand OA. According to the updated 2018 European Alliance of Associations for Rheumatology (EULAR) recommendations, first-line treatments include patient education, hand exercises, and assistive devices [3]. Topical non-steroidal anti-inflammatory drugs (NSAIDs) are the first choice for supplemental pharmacological treatment, while oral NSAIDs may be used for short-term pain relief. Intra-articular glucocorticoids injections may be considered for patients with painful interphalangeal joints, and surgery may be offered when other treatment options have not provided sufficient pain relief [3].

National healthcare policies recommend that OA treatment should mainly be delivered in primary care [4]. However, research has revealed that people with hand OA have poor access to recommended treatments in this setting [5,6]. This is supported by findings from a Norwegian trial, where only 21 ​% of participants had received recommended non-pharmacological treatment before being referred by their general practitioner for surgical consultation due to thumb-base OA [7]. The results also indicate a gap between recommended and available treatments for people with hand OA and highlights an urgent need for a tool to monitor and evaluate initiatives aimed at improving future deliverance of high-quality care.

High quality care is defined as clinically effective, safe, and tailored to the individual, delivered to all users of any health service in all phases of care [8]. Quality indicators (QIs) are used to monitor and evaluate current care and thereby to improve quality of care. A QI is “a measurable element of practice performance for which there is evidence or consensus that it can be used to assess the quality, and hence change in the quality, of care provided” [9]. QIs assess how effectively health services improve the likelihood of achieving desired health outcomes. They encompass three key aspects: the structure of care (the settings where care is delivered), the process of care (the actions taken during care delivery), and the outcomes of care (the results achieved) [10]. They may be used to inform authorities and stakeholders; serve as tools for internal quality improvement; and assist patients in choosing service providers [11].

There are several existing QI-sets for OA care [12,13]. One is the Health Care Quality Indicators for OA launched by The EUMUSC.NET project in 2013 [14], containing twelve general statements about care for people with OA. Another is the OsteoArthritis Quality Indicator (OA-QI) questionnaire [15], developed in 2010 in Norway to assess patient-reported quality of OA care. This set of 17 QIs was based on published QIs identified through a literature review, and thereafter discussed with two expert panels and patient representatives. The set was revised in 2015, following feedback from researchers and patient research partners (PRPs) and reduced to 16 items. In 2018, the revised version, OA-QI v.2, was assessed for reliability, validity, responsiveness and interpretability with good results [16]. This set is the most up-to-date version across validated sets of quality indicators in OA and is one of the few that incorporates the highly valued patient-reported perspective [17,18]. Importantly, none of the existing QI-sets are developed specifically to assess the quality of care for people with hand OA [17].

Given the relevant and contemporary design of the OA-QI v.2. questionnaire, the aim of this study was to adapt it for evaluating the quality of care for individuals with hand OA.

## 2 Methods

### 2.1 Design

**Fig. 1:**
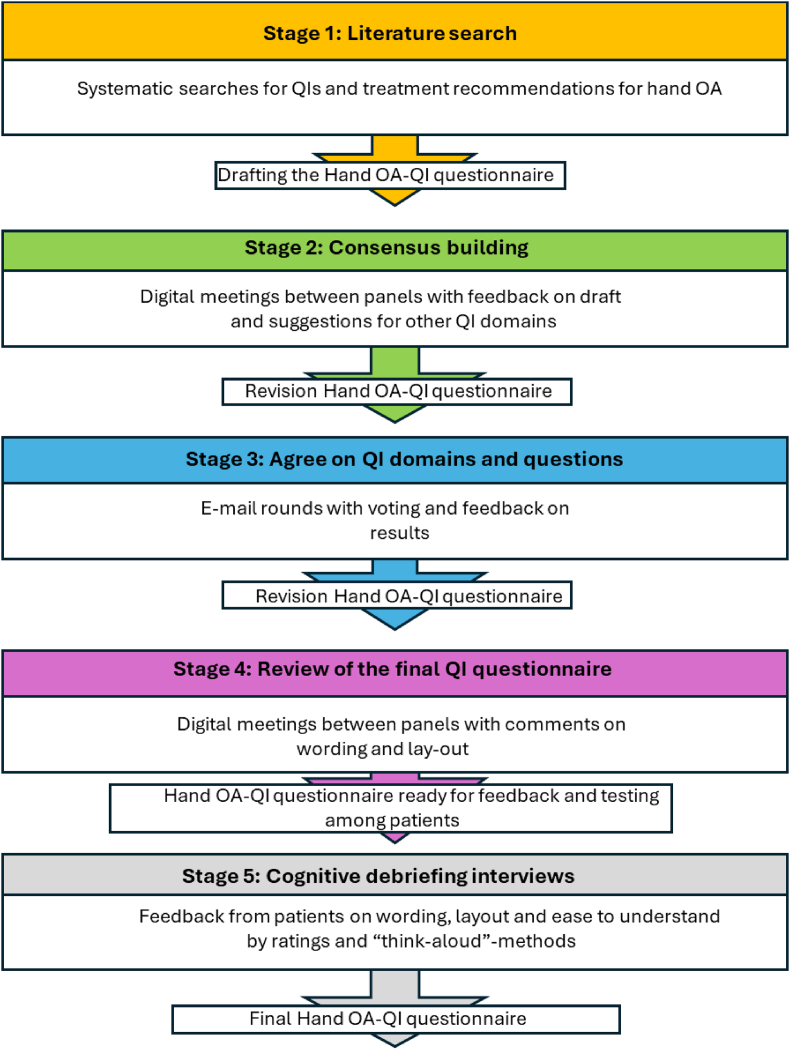
Flowchart illustrating the stages to develop the Hand OA-QI questionnaire.

We adapted the English version of the OA-QI v.2 questionnaire for use in hand OA care using the RAND Corporation/University of California Los Angeles (Rand/UCLA) appropriateness method [19]. This method integrates evidence reviews, multidisciplinary expert panel-meetings and repeated anonymous ratings for consensus building [19]. The process was conducted in five stages, with the first four focusing on reaching consensus on a set of QIs provided in both the Norwegian and English languages. In the fifth stage, we conducted debriefing cognitive interviews with Norwegian and UK individuals with hand OA to optimize the wording and lay-out of the QI-questionnaire (Fig. 1).

Two separate expert panels were formed. The first, also called the project group, was responsible for administering background materials and organizing the various stages and comprised one patient research partner and eight researchers and/or occupational- and physical therapists from four European countries: Norway, Sweden, the UK, and Austria. All members except for the project leader (IK) also participated in the consensus process, including the voting process in stage 3 described below.

The second expert group, which only participated in the consensus process, comprised two PRPs with hand OA, and eight professionals from various backgrounds such as physicians, rheumatologists, occupational therapists, researchers and a surgeon. Members represented different nationalities, including the UK, Norway, Netherland and France. Participants were selected using a strategic sampling approach, considering their prior experience, work, and positions within the field. In both panels, the goal was to ensure a diverse and balanced panel composition in terms of nationalities, level of care, clinical versus research experience, and a range of professional versus patient viewpoints.

### 2.2 Development of items

We conducted a systematic literature search in the Medline, Embase, CINAHL, and AMED databases to identify QI sets for OA published after 2015, when the OA-QI questionnaire was last revised. The identified papers were uploaded to the software RAYYAN and independently screened by the first and last author.

To support the selection of indicators, we also summarized the following international treatment recommendations for hand OA: the 2018 update of the EULAR recommendations for the management of hand OA [3], the 2019 American College of Rheumatology/Arthritis Foundation Guideline for the management of OA of the hand, hip, and knee [20], the 2019 OARSI guidelines for the non-surgical management of knee, hip, and polyarticular OA [21], and the 2022 National Institute of Health and Care Excellence (NICE) guidelines for OA [22]. Additionally, project members working on an update of the systematic review that informed the 2018 EULAR recommendations provided the panels with an updated summary of evidence on the effectiveness of various treatments for hand OA [23]. Based on this information, the project group developed a first draft of the Hand OA-QI questionnaire, which included both original relevant QIs and suggestions for additional QIs or reformulations drawn from the treatment recommendations and new evidence to reflect hand OA care more specifically.

In stage 2, both the original OA-QI v.2 and the first draft of the Hand OA-QI questionnaire were emailed to both expert groups. During an online meeting with members from both panels, the summaries generated in stage one was presented, followed by a discussion where participants could suggest additional items and wording changes. The goal was to formulate potential indicators that could be answered with a simple “yes” or “no,” indicating whether the respondent had received the recommended treatment or not. Feedback from this first meeting was used by three members of the project group (DHB, YP and IK) to revise the first draft of the Hand OA-QI questionnaire. The updated version was then redistributed to all members of both expert groups prior to a second round of online panel discussions emphasizing contents, linguistics, and layout.

Stage 3 involved consecutive anonymous voting rounds among eligible panellists regarding which indicators to include. This was done using the Questback.com®-service, an online tool well-suited for this purpose and was administered by an assistant not otherwise involved in the study. The Questback® allowed participants to consecutively decide on the inclusion of eligible items by electronically selecting their responses in an online survey while also refining the wording of the indicators under consideration. After each round, the tool generated a blinded post-vote group summary of the results for the administrator. More specifically, panel members rated each potential indicator on Likert scales ranging from 1 to 9 (with 9 representing high degree of agreement) across four domains [24]*; importance* of the measured item, *scientific soundness* (validity), *usefulness* and *feasibility*. For each domain, median scores were calculated and the four domains were classified into three levels of appropriateness: 1) “appropriate”, defined as a panel median of 7–9 for all criteria, which resulted in inclusion in the final QI-questionnaire; 2) “uncertain”, defined as a panel median between ≥3 and ​< ​7 leading to inclusion in a subsequent voting round, and 3) “inappropriate”, defined as a panel median <3, which resulted in exclusion from further voting rounds. If consensus could not be reached for indicators classified as “uncertain” after repeating voting rounds, a yes/no vote was held and concluded if at least two-thirds of the participants agreed on an option [19]. Suggested linguistic changes were saved for usage in stage 4.

In stage 4*,* an online meeting with all panellists was organized to finalize the wording and layout of the questionnaire. At the same time, a Norwegian translation of the questionnaire (Hand OA-QI NOR) was prepared by members of the project group.

### 2.3 Cognitive debriefing interviews

In stage *5*, we conducted cognitive debriefing interviews with Norwegian and British participants, all of whom had hand OA, to ensure that potential end users understood the items in the Hand OA-QI questionnaire as intended by the developers [25]. In Norway, participants were recruited at Diakonhjemmet Hospital in Oslo and at Haugesund Rheumatological Hospital. The recruitment of participants in the UK was carried out through the University of Salford's research volunteers' database and social media (specifically the X and Linked In platforms). Purposive and diverse sampling was employed to adequately represent the experiences of the target population across different levels of severity of the condition, ensuring a comprehensive range of patient experiences are captured. This strategy included selecting participants who varied in terms of disease severity, demographic characteristics, and educational levels to inform the necessary revisions. All participants provided written informed consent.

All interviews were scheduled to last for up to 1 ​h and were conducted face-to-face in Norway and by phone in the UK. The interviews began with a “think-aloud” approach, where participants were asked to verbalize their thoughts about each item, including the layout and ease of understanding while completing their Hand OA-QI questionnaire [25]. Participants were asked structured questions about the questionnaire's instructions, recall period and responses. Finally, participants rated the ease of understanding each domain on a five-point scale where 1 reflected very easy to understand, and 5 very difficult to understand. Researchers also took notes during the interviews. In Norway, each participant received a standardized honorarium of €50/meeting for their participation. Participants in the UK were not reimbursed for their time, as the phone interviews did not incur any associated costs for them.

The project was conducted in accordance with the Helsinki declaration [26]. All invited participants were given the opportunity to ask questions about the project and the information collected before deciding to provide informed consent. Signed consent could be withdrawn at any time. Participants were encouraged to avoid discussing unrelated personal health issues during the interviews. Notes produced during the interviews were stored in a secure place until the analyses were completed, after which physical documents were shredded, and electronic copies of responses were permanently deleted. The project was approved by the data protection officer at Diakonhjemmet Hospital September 2022 (project number DS00656), and University of Salford Research Ethics Panel in September 2023 (Application ID: 13577).

### 2.4 Data analysis

Each cognitive debriefing interview was analysed descriptively and reviewed for instances where participants identified needs for clarifications regarding items, instructions, response choices, and recall period. The feedback was then discussed by the project group, and modifications to the QIs were made if three or more participants found a particular element notably difficult to understand (median score >2).

## 3 Results

### 3.1 Development of items stages 1–4

A total of 1670 papers were identified in the systematic literature search. After thorough review of the identified articles, no quality indicator instruments relevant to the management of hand OA in terms of quality of care were identified (Supplementary file 1).

During the initial meetings in stage 2, a throughout evaluation of the outcomes for which the item interventions should address was performed by the panels. Reflecting the literature and feedback from the PRPs, there was strong agreement that priority should be given to pain and function, as well as enhancing patients' overall knowledge of the disease or symptoms and their ability to remain in the workforce. Even if non-articular drivers of pain may be present, the panellists preferred base their decisions on evidence from randomized controlled trials in individuals with hand OA, specifically considering an intervention's accessibility, safety and effectiveness. However, the panellists acknowledged that the evidence regarding these aspects may be limited or applicable only to specific subgroups, such as individuals with thumb-base OA. It was emphasized that each individual indicator should assess only one dimension of care at a time, with feasible response options were suggested. Lastly, an introductory text was drafted.

Six additional indicators were suggested for inclusion in later voting rounds. These indicators covered the areas of work participation (n ​= ​1), patient education (n ​= ​1), orthosis (n ​= ​1), scheduled follow-up (n ​= ​1), pharmacological interventions (n ​= ​1), and thermal modalities (n ​= ​1). As a result, the total number of potential indicators at the end of Stage2 increased to 22 (Table 1).

**Table 1:**
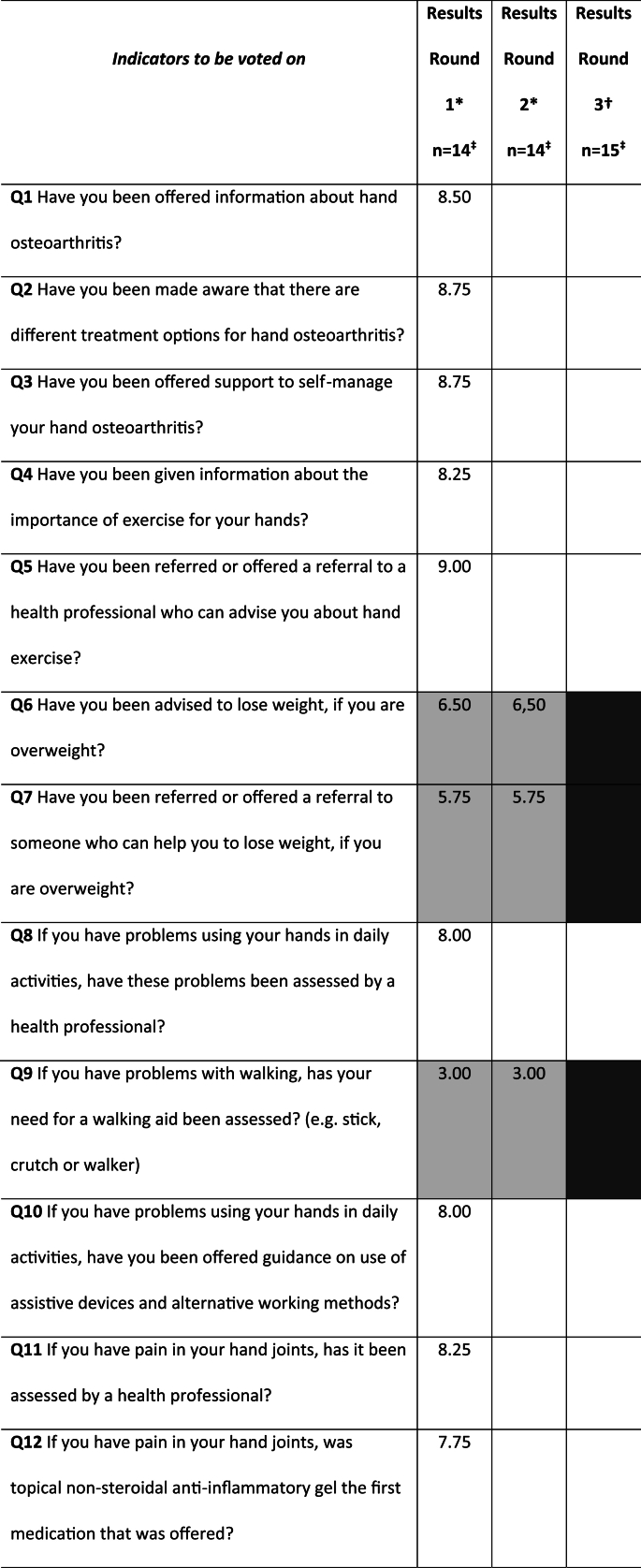

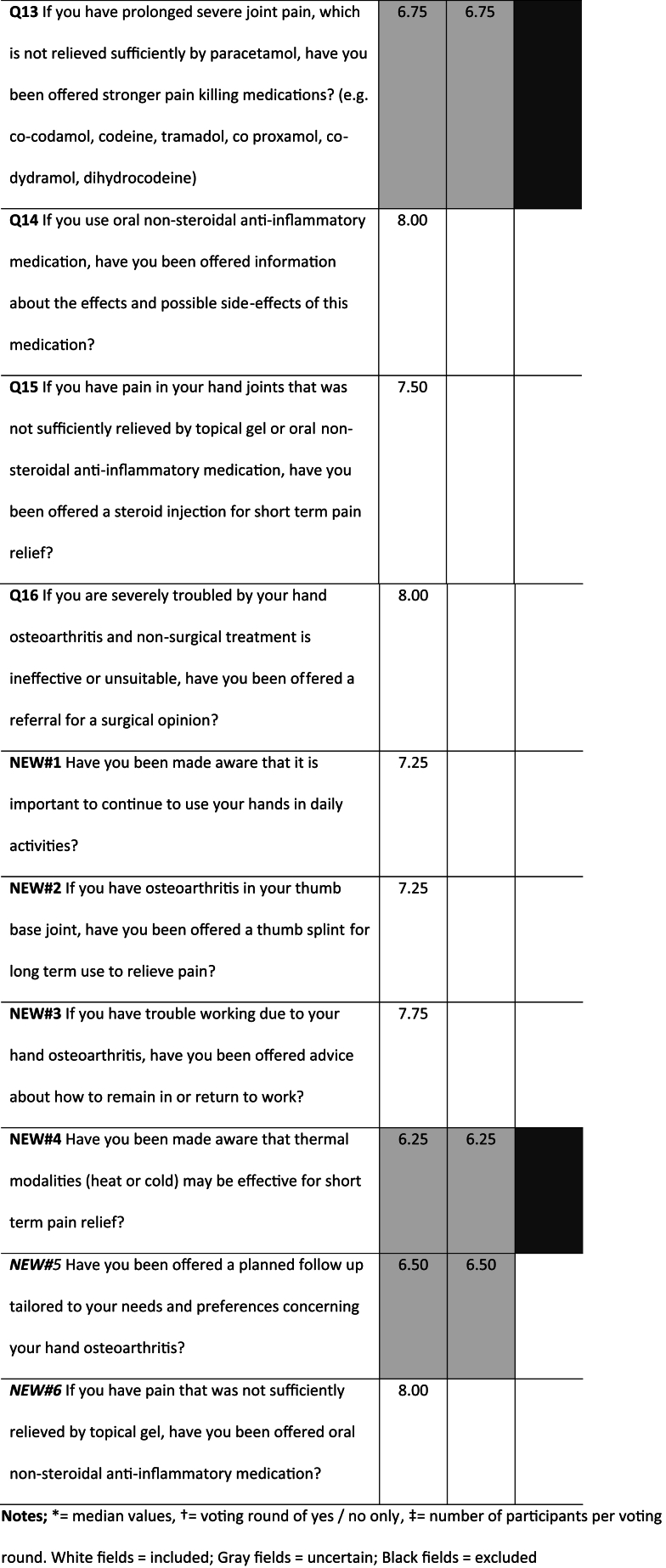
Results from the voting rounds of all eligible quality indicators in stage 3 (eligible participants N ​= ​18).

In stage 3, consensus on the items to be included in the Hand OA-QI questionnaire was reached after three rounds of voting (Table 1). Panellist participation rates were N ​= ​13 (72.2 ​%) in both the first and second rounds, and N ​= ​15 (83.3 ​%) in the third round. In the first round of voting, six indicators were categorized as uncertain based on their median scores (Table 1). Consequently, we repeated the voting for these six draft-indicators alone, but the results remained unchanged. During discussions after the two first voting rounds, it was noted that the indicators related to exercises (items 4 and 5) were overlapping, thereby increasing response time and adding to end-user burden. A pre-defined third round, using only “yes” and “no” options, was then conducted (Table 1) to agree upon the six indicators not concluded in previous rounds, as well as merging of the two items relating to exercise. In this round, more than two-thirds of the participants agreed that the original QI OA v.2. items related to weight loss advice, weight loss help, walking aids and stronger pain killers should be excluded*,* along with the item concerning thermal treatment modalities suggested in stage 2. However, the item addressing a scheduled follow-up was retained. In the same voting round, 77.7 ​% (n ​= ​14) supported merging the two exercise indicators into a single item and rewording it. As a result, the final draft of the new Hand OA-QI questionnaire comprised 16 items, addressing patient education (n ​= ​3), self-management support (n ​= ​1), work-related advice (n ​= ​1), exercises (1), patient assessments (2), orthoses and assistive devices (2), pharmacological interventions (4), surgical consultation (1) and patient follow-up (1). In addition, an introduction specifying that respondents may have received or acquired information from multiple sources, including social media and mobile applications was included.

In stage 4, both the Norwegian translation and the original English versions of the questionnaire were reviewed by panellists proficient in the respective languages for their wording, with no new comments being made.

### 3.2 Cognitive debriefing interviews

A total of 28 participants took part in the cognitive debriefing interviews in stage 5, with 13 and 15 participants being interviewed in Norway and the UK, respectively (Table 2). Most participants found their respective translations of the questionnaire straightforward to complete. However, eight patients (seven in Norway, one in the UK) commented on the meaning of “support” in question 4, as the same word could refer to either physical (i.e., an orthosis) or psychosocial support. The median clarity score for this question was 2. Still, since participants did not offer any suggestions for improvement, the wording was not changed. Four participants requested clarification for the term “NSAIDs”. The median clarity score for all questions involving NSAIDs was 1. Based on this feedback, examples of topical and oral NSAIDs were listed in questions number 11 and 12.

**Table 2:** Characteristics of participants included in the cognitive debriefing interviews in stage 5.

Characteristics	Norwegian sample (N ​= ​13)	English sample (N ​= ​15)
Age, range	48–81	34–77
Sex, female (%)	11 (84.6)	10 (66.6)
OA duration (years), median (IQR)	9 (3–12)	7 (2–10)

*Abbreviations:* OA, osteoarthritis; IQR, inter-quartile range.

Four Norwegian participants recommended rewording question number 15, as its multiple negations made it difficult to understand.

Finally, informants provided feedback on the layout and response options in the questionnaire. The initial version included specific examples of information sources in the introduction section. However, since most participants found these examples redundant or likely to become outdated, they were removed.

Most participants felt it was inappropriate to specify a limited time frame for recalling items, given the variability in people’ disease stages and the contexts in which the QI set might be applied. Five participants suggested adding a response option for cases where certain domains are not feasible or applicable to the respondent, specifically in questions 8, 9, 11, 12, and 13. Following further discussions, the expert panel members recognized that the response options in the hand OA questionnaire versions used during the cognitive debriefing interviews did not adequately address certain scenarios and needed modifications before further use. Therefore, the response option “not taking such drugs” was replaced by “not applicable” in questions 11 and 12. The response option “no such problems” remained unchanged for questions 8 and 9. The complete Norwegian and UK versions of the questionnaire are found in Supplementary Tables 1 and 2, respectively.

## 4 Discussion

In this study, we have adapted the OA-QI v.2.-questionnaire into a 16-item tool designed to assess the quality of care for individuals with hand OA; the Hand OA-QI questionnaire. The new instrument is grounded in research evidence, multidisciplinary expert panel-meetings, repeated anonymous ratings, and cognitive debriefing interviews with people living with hand OA.

Symptomatic hand OA is common among working-age individuals and whose symptoms may interfere with their professional lives [[27], [28], [29], [30]]. Previous research highlights the importance of prioritizing timely and work-relevant assessments in hand OA care, as well as supporting work participation tailored to each patient's individual needs [[31], [32], [33]]. These aspects were not addressed in the original OA-QI v.2 instrument. The inclusion of an item specifically designed to assess work-related evaluation in the Hand OA-QI questionnaire thereby introduces a novel dimension that reflects the real-world challenges faced by patients.

Two items from the OA-QI v.2 instrument, which assessed interventions relatable to weight loss, were not included in the Hand OA-QI questionnaire. While lifestyle interventions, such as weight management, are key components of OA care [31] the specific effects of weight loss on hand OA symptoms have yet to be thoroughly investigated. However, weight can impact overall comorbidity burden and quality of life [34,35], which suggests that advocating for a healthy weight could still be an important aspect of OA management for some individuals [36].

The evidence supporting the effectiveness of stronger pain medications, such as codeine and tramadol, in improving pain and function in hand OA is limited [3]. The Hand OA-QI questionnaire therefore includes only NSAIDs as first and second-line pharmacological interventions. Glucocorticoids are frequently used in clinical practice for hand OA, yet the evidence supporting them is not convincing and recommendations vary by guidelines [3,4,20,21,37]. Despite recently demonstrated short-term efficacy of Prednisolone among patients with signs of inflammation [38], the long-term outcomes and use among the broader patient group should be further explored to understand its role [39]. Recent research shows that Methotrexate may offer pain relief for patients with concurrent synovitis [40], however, this treatment is not recognized in the existing hand OA guidelines, and consequently, is not featured in the Hand OA-QI questionnaire.

Patients with hand OA may have limited access to recommended treatments [5,41] and have expressed the need for easily accessible self-management interventions [42]. Recently, technological advancements have enabled traditional face-to-face health care services in knee OA to be delivered through digital platforms (eHealth), particularly through smartphone or mobile health technology (mHealth) [43]. Despite the current scarcity of mHealth solutions specifically targeting hand OA, two pioneering studies recently demonstrated the feasibility of mHealth in managing hand OA [44,45]. Future studies are warranted to explore the impact of mHealth in hand OA care deliveries, for which the Hand OA-QI questionnaire is designed to accommodate by recognizing digital media and related channels as valid sources of information, thus, futureproofing the tool for evolving treatment models.

In addition to measuring the quality of current care, the indicators in the Hand OA-QI instrument may serve a normative function by clearly defining best practices in the treatment of hand OA. This could have an educational benefit for patients and care-providers, guiding them to recommended treatments and thereby accelerating the implementation of evidence-based hand OA care. In this way, the questionnaire could assist in achieving the goals outlined in the EULAR strategy 2018–2023; to deliver preeminent comprehensive quality of care frameworks for the management of people with rheumatic and musculoskeletal diseases, and to address the observed lack of timely access to sufficient care and specialists identified by the EULAR manifesto 2024–2029 [46].

Implementing the current QI-set into clinical practice and research is still constrained by the lack of thorough psychometric testing. However, the development process itself supports the validity of the Hand OA-QI questionnaire. First, previous research has shown that the web-based Rand/UCLA Appropriateness Method is a reproducible and preferred approach for our purpose. It combines the best available evidence with open discussions and standardized anonymous expert judgements to develop consensus [47,48]. Second, the broad composition of panels and high number of PRPs from different countries enabled us to incorporate a variety of viewpoints into discussions and anchor the instrument's clinical transparency and scientific integrity [19,48]. However, the authors acknowledge a potential bias in stage 3 due to incomplete panel participation in each voting round. Anonymity prevents identifying whether the same participants abstained, making it unclear whether any imbalances in perspectives occurred. Third, all eligible indicators were grounded in leading guidelines and the latest updates on the efficacy of various treatments for hand OA, ensuring alignment with international standards of OA care. Finally, during cognitive debriefing interviews, people with hand OA found the items understandable and acceptable. Nonetheless, the questionnaire still needs to be tested and applied to a broader and more diverse patient population to fully establish its validity.

This testing should also assess key aspects of reliability such as internal consistency and test-retest reliability, as well as responsiveness, which refers to the instrument's sensitivity to change over time [49]. This process is currently underway, with testing being conducted in the PICASSO trial [50].

In conclusion, we have successfully adapted the OA-QI v.2.-questionnaire into a 16-item tool for hand OA care specifically designed to assess the quality of care for individuals with hand OA, available in both Norwegian and English languages, also deemed easy to understand by patients involved in the development process. The new instrument has the potential to enhance the use of evidence-based interventions in hand OA care, thereby improving timely access to recommended treatments. Evaluation of the psychometric properties of the instrument in both English and Norwegian languages is necessary before they can be fully implemented in clinical practice.

## Author contributions

**(1) Substantial contributions to the conception or design of the work;** DHB, YP, NØ and IK **or the acquisition, analysis, or interpretation of data for the work;** DHB, YP, RB, FB, MB, TB, BSC, KD, JE, NG, IKH, CI, MK, HL, MJPFR, TS, ATT, NØ and IK **AND (2) Drafting the work** DHB, YP, IK **or reviewing it critically for important intellectual content;** DHB, YP, RB, FB, MB, TB, BSC, KD, JE, NG, IKH, CI, MK, HL, MJPFR, TS, ATT, NØ and IK **AND (3) Final approval of the version to be published;** DHB, YP, RB, FB, MB, TB, BSC, KD, JE, NG, IKH, CI, MK, HL, MJPFR, TS, ATT, NØ and IK **AND (4) Agreement to be accountable for all aspects of the work in ensuring that questions related to the accuracy or integrity of any part of the work are appropriately investigated and resolved** DHB, YP, RB, FB, MB, TB, BSC, KD, JE, NG, IKH, CI, MK, HL, MJPFR, TS, ATT, NØ and IK**. DHB takes responsibility for the integrity of the work as a whole, from inception to finished article.**

## Role of the funding source

The authors confirm that the funding source (EULAR) did not involve in decisions concerning the study design, collection, analysis and interpretation of data; in the writing of the manuscript; nor in the decision to submit the manuscript for publication.

## Declaration of competing interest

DHB reports that his work in the current project was funded by the 10.13039/501100008741EULAR 10.13039/100016179HPR research grant through his institution. FB reports consulting fees, support and honoraria from Grunenthal, GSK, Eli Lilly, 10.13039/100004336Novartis, 10.13039/100004319Pfizer, 10.13039/501100011725Servier, 4P Pharma, Viatris, 10.13039/100012895Zoetis and 10.13039/501100014812Nordic Pharma, and board participation in 10.13039/100004325AstraZeneca, 10.13039/501100013671Sun Pharma and Nordic Bioscience outside the current work. KD is part funded by the 10.13039/501100000272National Institute for Health and Care Research (NIHR) Applied Health Research Collaboration (ARC) West Midlands (NIHR 200165) and the Birmingham Biomedical Research Centre (BRC). KD is also an NIHR Senior Investigator (ID NIHR 205031). The views expressed are those of the author(s) and not necessarily those of the NIHR or the Department of Health and Social Care. IKH reports honoraria from Novartis, GSK, Grünethal and Abbvie outside the current work. MK reports consulting fees and honoraria from the IMI-APPROACH, 10.13039/100018286Dutch Arthritis Society, Wolters Kluwer, Springer Verlag, as well as board fees from 10.13039/100004319Pfizer, 10.13039/100011110UCB, 10.13039/100022756CHDR, GSK, 10.13039/100004336Novartis and Peptinov paid to her institution. She is also a OARSI board member, EULAR council member and president of Dutch society of Rheumatology. MJPFR reports consulting fees from Loci Orthopaedics outside the current work. TS reports grant support from Roche and personal fees from 10.13039/100006483AbbVie, 10.13039/100004337Roche, 10.13039/100004339Sanofi, Takeda, and 10.13039/100004336Novartis, outside the submitted work. YP, RB, TB, JE, NG, HL, ATT, NØ and IK report nothing to declare.
