# Supplementary material for: Development of quality indicators for hand osteoarthritis care – Results from an European consensus study
Source: Osteoarthr Cartil Open. 2025 Feb 5;7(1):100578. doi: 10.1016/j.ocarto.2025.100578 (PMC11849605; doi:10.1016/j.ocarto.2025.100578)
Supplement: Multimedia component 1 [file mmc1.docx]

SPØRSMÅL OM BEHANDLINGEN DU HAR MOTTATT FOR DIN HÅNDARTROSE E

Det er flere ulike behandlingsalternativer for håndartrose. Ved å svare på spørsmålene nedenfor hjelper du oss med å bedre behandlingen for personer med håndartrose. For hvert spørsmål ber vi deg sette ett kryss i en av de tre rutene for å svare på hvilken behandling, informasjon eller støtte du har mottatt eller funnet frem til i løpet av de siste månedene. Kilden kan være helsepersonell, eller kilder du anser som pålitelige innen media, internett, sosiale medier, apper for smarttelefon, pasientorganisasjoner, eller familie og venner.

|  |  | **Ja** | **Nei** | **Husker ikke** |
| --- | --- | --- | --- | --- |
| 1 | Har du fått informasjon om håndartrose? |  |  |  |
| 2 | Har du blitt gjort oppmerksom på at det er viktig å bruke hendene i daglige aktiviteter? |  |  |  |
| 3 | Har du blitt gjort oppmerksom på at det finnes ulike behandlingsalternativer for håndartrose? |  |  |  |
| 4 | Har du blitt tilbudt støtte for å mestre din håndartrose? |  |  |  |
| 5 | Har du fått veiledning i hvordan du skal trene hendene dine? |  |  |  |
|  |  | **Ja** | **Nei** | **Ingen slike problemer** |
| 6 | Dersom du har problemer med å bruke hendene i daglige aktiviteter, har disse problemene blitt vurdert av helsepersonell? |  |  |  |
| 7 | Dersom du har problemer med å bruke hendene i daglige aktiviteter, har du fått veiledning i bruk av hjelpemidler og alternative arbeidsmetoder? |  |  |  |
|  |  | **Ja** | **Nei** | **Ikke aktuelt** |
| 8 | Dersom du har artrose i tommelens rotledd, har du fått tilbud om tommelstøtte/tommelortose for bruk over tid for å lindre smertene? |  |  |  |
| 9 | Dersom du har problemer med å arbeide på grunn av din håndartrose, har du fått råd om hvordan du kan fortsette å være i arbeid eller komme tilbake i arbeid? |  |  |  |
|  |  | **Ja** | **Nei** | **Ingen smerter** |
| 10 | Dersom du har smerter i fingerleddene, har disse blitt vurdert av helsepersonell? |  |  |  |
|  |  | **Ja** | **Nei** | **Ingen smerter / ikke aktuelt** |
| 11 | Dersom du har smerter i fingerleddene, var smertedempende NSAID *gel* eller *krem* (for eksempel Ibux gel eller Voltarol gel) det første medikamentet du ble tilbudt for å dempe smertene? |  |  |  |
| 12 | Om smertedempende gele eller krem ikke ga ønsket effekt, har du da fått tilbud om NSAID-*tabletter* (f.eks. Ibux, Ibuprofen eller Voltaren)? |  |  |  |
|  |  | **Ja** | **Nei** | **Tar ikke slike medisiner** |
| 13 | Dersom du bruker NSAID-*tabletter*, har du fått informasjon om virkning og mulige bivirkninger av disse? |  |  |  |
|  |  | **Ja** | **Nei** | **Ingen slike smerter** |
| 14 | Dersom du har smerter i fingerleddene og ikke har oppnådd tilstrekkelig lindring med NSAID gel, krem eller tabletter, har du da fått tilbud om kortisoninjeksjon for å gi kortvarig smertelindring? |  |  |  |
|  |  | **Ja** | **Nei** | **Ingen store plager** |
| 15 | Dersom du er veldig plaget av din håndartrose og annen behandling ikke har gitt ønsket effekt, har du fått tilbud om henvisning til kirurgisk vurdering? |  |  |  |
|  |  | **Ja** | **Nei** | **Husker ikke** |
| 16 | Har du fått tilbud om en planlagt oppfølgning tilpasset dine behov? |  |  |  |
