# Supplementary material for: Development of quality indicators for hand osteoarthritis care – Results from an European consensus study
Source: Osteoarthr Cartil Open. 2025 Feb 5;7(1):100578. doi: 10.1016/j.ocarto.2025.100578 (PMC11849605; doi:10.1016/j.ocarto.2025.100578)
Supplement: Multimedia component 2 [file mmc2.docx]

## Questions on the management of your hand osteoarthritis

There are several different treatment alternatives for hand osteoarthritis. By answering the questions below, you will help us improve the care for people with hand osteoarthritis. For each question, please tick off one of the boxes provided to answer what treatment, information, advice or support you have received or acquired for your hand osteoarthritis in the past _ months.
The source of information may be health professionals, mainstream media, internet, social media, mobile apps, patient organizations, family and friends or expert patients / peer-to-peer education.

|  |  | **Yes** | **No** | **Don’t  remember** |
| --- | --- | --- | --- | --- |
| 1 | Have you been offered information about hand osteoarthritis? | □ | □ | □ |
| 2 | Have you been made aware that it is important to continue to use your hands in daily activities? | □ | □ | □ |
| 3 | Have you been made aware that there are different treatment options for hand osteoarthritis? | □ | □ | □ |
| 4 | Have you been offered support to self-manage your hand osteoarthritis? | □ | □ | □ |
| 5 | Have you been offered guidance on how to exercise your hands? | □ | □ | □ |
|  |  | **Yes** | **No** | **No such  problems** |
| 6 | If you have problems using your hands in daily activities, have these problems been assessed by a health professional? | □ | □ | □ |
| 7 | If you have problems using your hands in daily activities, have you been offered guidance on use of assistive devices and alternative working methods? | □ | □ | □ |
| 8 | If you have osteoarthritis in your thumb base joint, have you been offered a thumb splint for long term use to relieve pain? | □ | □ | □ |
| 9 | If you have trouble working due to your hand osteoarthritis, have you been offered advice about how to remain in or return to work? | □ | □ | □ |
|  |  | **Yes** | **No** | **No pain** |
| 10 | If you have pain in your hand joints, has it been assessed by a health professional? | □ | □ | □ |
|  |  | **Yes** | **No** | **Not applicable** |
| 11 | If you have pain in your hand joints, was topical non-steroidal anti-inflammatory gel the first medication that was offered? | □ | □ | □ |
| 12 | If you have pain that was not sufficiently relieved by non-steroidal anti-inflammatory topical gel, have you been offered oral non-steroidal anti-inflammatory medication? | □ | □ | □ |
|  |  | **Yes** | **No** | **Not taking such drugs** |
| 13 | If you use oral non-steroidal anti-inflammatory medication, have you been offered information about the effects and possible side-effects of this medication? | □ | □ | □ |
|  |  | **Yes** | **No** | **No such pain** |
| 14 | If you have pain in your hand joints that was not sufficiently relieved by topical gel or oral non-steroidal anti-inflammatory medication, have you been offered a steroid injection for short term pain relief? | □ | □ | □ |
|  |  | **Yes** | **No** | **Not severely troubled** |
| 15 | If you are severely troubled by your hand osteoarthritis and non-surgical treatment is ineffective or unsuitable, have you been offered a referral for a surgical opinion? | □ | □ | □ |
|  |  | **Yes** | **No** | **Don’t  remember** |
| 16 | Have you been offered a planned follow up tailored to your needs and preferences concerning your hand osteoarthritis? | □ | □ | □ |
