# Supplementary material for: Development of quality indicators for hand osteoarthritis care – Results from an European consensus study
Source: Osteoarthr Cartil Open. 2025 Feb 5;7(1):100578. doi: 10.1016/j.ocarto.2025.100578 (PMC11849605; doi:10.1016/j.ocarto.2025.100578)
Supplement: Multimedia component 3 [file mmc3.docx]

**Supplementary file 1_ Bordvik et al.**

**Literature searches ahead of the development of quality indicators for hand osteoarthritis (OA):**

*Purpose:* To identify studies presenting quality indicators relevant to hand OA, published until April 4, 2024.

*Eligibility criteria*: I.e., the extent to which the identified studies were in English or Scandinavian languages, providing the development or presentation of quality indicators addressing processes of care in hand OA, published after 2015.

*Search strategy*: The literature searches were conducted in the Medline, Embase, CINAHL, and AMED databases, using various key-words and MESH-terms, i.e., osteoarthritis, hand osteoarthritis, quality/standard of (health) care or practice guideline – searching across headlines, keywords, titles, abstracts, full-texts, original title, name of substance word, subject heading word, floating sub-heading word, keyword heading word, organism supplementary concept word, protocol supplementary concept word, rare disease supplementary concept word, unique identifier and synonyms.

*Results:* On April 19. 2022, the searches yielded 1670 papers after the removal of duplicates, all of which were uploaded to Rayyan for further screening. Two members of the project group (DHB, IK) independently screened the results by i.e., titles and abstracts, categorizing each paper as “include”, “exclude” or “uncertain” before an unblinded discussion with further assessment of potential articles classified different among the evaluators or as “uncertain”. If consensus was not met, a third project group member (ATT) was available for consultation. No studies were included. On January 25, 2023, an updated search was conducted, for which the same process was repeated for the 446 studies identified, with no included in the study.
